# Four Decades of HIV Antibody Screening in the U.S. Military: A Review of Incidence and Demographic Trends, 1990–2024

**Published:** 2025-04-20

**Authors:** Bulbulgul Aumakhan, Angelia A. Eick-Cost, Gi-Taik Oh, Shauna L. Stahlman, Robert Johnson

**What are the new findings?**

From 1990 through 2024, over 46 million tests for HIV antibodies were conducted among active, Guard, and reserve members of the U.S. Armed Forces, and 11,280 (24.3 per 100,000 persons tested) were diagnosed with HIV. Male service members comprised 96.3% of all HIV infections. The total rate of new HIV diagnoses declined over the period of surveillance, with the steepest decline in the first decade. Overall rates stabilized in 1997, but differences persist between different age and racial and ethnic population groups. New HIV diagnoses have risen among male service members under age 30 years, with non-Hispanic Black service members bearing the highest burden, while Hispanic service members demonstrating the largest relative increases. Since 1997, rates in all racial and ethnic groups have more than doubled for those under age 25 years; for Hispanic service members, the increase was nearly 10-fold.

**What is the impact on readiness and force health protection?**

Rising HIV infection rates among male U.S. service members under age 30 years underscores the need for targeted and enhanced prevention efforts to sustain progress and mitigate marked and increasing differences between specific populations and age groups. Given the impact of HIV on force readiness, optimization of screening strategies, including indications-based testing after service entry, could improve the effectiveness and value of current screening efforts. The HIV-antibody screening program remains an important element of force health protection.

Since 1985 the U.S. military has conducted mandatory HIV antibody screening of active and reserve members of the Armed Forces. ^1 , 2^ By 1990, all service members had been tested at least once, and routine screening primarily detected recent HIV infections. ^3^ *MSMR* began publishing summaries of HIV rates and trends in the U.S. military 10 years after screening began, in 1995. ^4^

In the initial years, routine screening detected both prevalent and incident HIV infections, with the rate of new HIV diagnoses among active duty U.S. Army members reported as high as 283 cases per 100,000 persons tested in 1985-1986. ^4^ With Department of Defense (DOD) policies barring HIV-positive individuals from entering or serving in the military, initial control efforts led to a precipitous drop in the rate of new HIV diagnoses in the active component, to approximately 30 cases per 100,000 individuals during the 1990s. ^3 , 5^ By the 2000s, overall rates of new HIV diagnoses continued to decline, albeit slowly, subsequently stabilizing during the following 2 decades, within a range of 20-25 new cases per 100,000 individuals tested, or approximately 350 to 400 new infections annually. ^6^ This marked decline and stabilization within a decade and a half evidences the success of the U.S. military HIV program in controlling HIV spread and maintaining low rates of infections within its ranks. ^5 , 6^

In 2004, the DOD adjusted the standard HIV testing interval from annual to biennial. ^7^ Reflecting evolving screening practices and shifts in the epidemiological profile of HIV cases, in 2005 *MSMR* began summarizing HIV rates and trends starting from 1990. In 2011, *MSMR* shifted to reporting summary HIV rates for the most recent 5.5 years, reflecting the stabilization of seropositivity rates and advancements in HIV treatment that established HIV as a chronic, manageable condition. ^7 , 8^

Despite significant efforts to further reduce HIV incidence, including the introduction and scaling of pre-exposure prophylaxis (PrEP) in 2012, the repeal of the “Don't Ask, Don't Tell” (DADT) policy in 2011, and test-and-treat initiatives aligned with the 2019 “Ending the HIV Epidemic” initiative, annual rates have continued to show little to no annual declines. To better understand the nature of new infections in the U.S. military, this retrospective analysis examined cases and trends in greater depth to 1) identify and describe total HIV antibody seropositivity rates from 1990 to 2024, with stratification by demographic characteristics, and 2) identify and characterize potential shifts in the epidemiological profile of HIV cases during the course of the evolving HIV epidemic.

## Methods

The population of interest for this study included all individuals screened for HIV antibodies while serving in the active, reserve and National Guard components of the U.S. Army, Navy, Air Force, Marine Corps, and Coast Guard. The surveillance period covered January 1, 1990 through December 31, 2024. Data analysis followed the case definition and incidence rules established by the Armed Forces of Health Surveillance Division (AFHSD) for HIV surveillance in the U.S. military. Laboratory testing methods and decision-making algorithms for identifying HIV infection are standardized and have been described in detail previously. ^1 , 6^

All individuals tested through U.S. military medical testing programs were ascertained from the Department of Defense Serum Repository (DODSR) specimens accessioned to the Defense Medical Surveillance System (DMSS). Annual HIV diagnosis rates reflect new infections identified among service members tested each calendar year, calculated as the number of HIV antibody seropositive cases per 100,000 persons. An individual was counted once per calendar year if that person was tested for HIV during that year. Due to incomplete or unavailable specimen and HIV testing results for the Coast Guard before 1996 and Air Force prior to 2006, these years were excluded from the relevant analyses.

For the descriptive characterization of HIV cases, distributions by demographic and military factors, such as age, race, service branch, and occupation, were examined. To facilitate identification and characterization of changes in the epidemiological profile of HIV cases within the evolving HIV epidemic, the 35-year surveillance period was divided into distinct phases. Key events and historical milestones that shaped the efforts to control HIV both nationally and within the U.S. military over the course of HIV surveillance were considered according to 4 phases: 1) the early epidemic, 1990-1995, 2) implementation of highly active antiretroviral therapy (HAART), 1996-2005, 3) expanded testing and prevention, 2005-2013, and 4) the modern era: “Ending the HIV Epidemic,” 2014-present **(Table 1)** .

**TABLE 1.** Phases of the HIV Epidemic, 1990–Present

Phase	Time Period	Description
Early Epidemic	1990–1995	This period is characterized by growing recognition of the HIV epidemic, streamlining of HIV testing and screening algorithms, early prevention efforts, and limited treatment options and effectiveness.
HAART	1996–2005	This period is distinguished by the introduction and widespread availability of highly active antiretroviral therapy (HAART) in 1996, and transformed HIV from a fatal disease to a manageable chronic condition.
Expanded Testing and Prevention	2005–2013	This period followed DOD adjustment of mandatory screening frequency to biennial in 2004, CDC expansion of routine testing guidelines in 2006 to include all adults, introduction of PrEP in 2012, and repeal of “Don't Ask, Don't Tell” policy in 2011.
Modern Era: *Ending the HIV Epidemic*	2014–Present	This period is marked by the launch of the “Ending the HIV Epidemic” initiative in 2019 and focuses on PrEP to support “Undetectable=Untransmittable” (U=U) principle.

Abbreviations: HIV, human immunodeficiency virus; HAART, highly active antiretroviral therapy; DOD, Department of Defense; CDC, Centers for Disease Control and Prevention; PrEP, pre-exposure prophylaxis.

HIV rate analyses of age-related trends for male service members were restricted to 1997-2024, as male rates stabilized after 1997. This restriction minimized the influence of early surveillance fluctuations. Since rates for the female population followed a more consistent trajectory, no such restriction was applied. In addition, due to low female case counts, female data were aggregated into 10-year age groups, to improve graphic interpretation. Where appropriate, further aggregation was applied to charting both male and female data if no notable variations were observed between grouped categories.

## Results

### Total, branch of service, and component seropositivity rates

**FIGURE 1.**
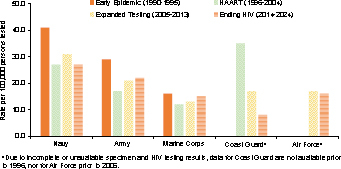
HIV Seropositivity by Service and Epidemic Phase, Active Component, U.S. Armed Forces, 1990–2024

From January 1990 through December 2024, a total of 46,409,929 annual tests for HIV antibodies were conducted among active, Guard, and reserve service members of the U.S. Armed Forces. During this 35-year surveillance period, 11,280 service members were diagnosed with HIV, yielding a crude total seropositivity rate of 24.3 per 100,000 persons tested **(Table 2)** . Among the service branches, the Navy had the highest overall rate, at 30.3 cases per 100,000 persons tested, followed by the Army, at 26.3 cases per 100,000 persons tested, while the Marine Corps, Air Force, and Coast Guard had lower rates, averaging about 15 new cases per 100,000 persons tested **(Figure 1)** . After the initial decline during the earliest phase of the epidemic, rates of new HIV diagnoses in the Army and Marine Corps gradually increased during subsequent phases. Rates were relatively similar in the early stages of the epidemic but diverged by the current Ending the HIV Epidemic phase. The highest rates were in the reserve component, followed by the Guard, with lowest rates in the active component **(Table 2)** .

**TABLE 2.** Epidemiological Profiles of Incident HIV Cases, U.S. Armed Forces, 1990–2024

Characteristics	Overall 1990-2024			Early Epidemic 1990-1995			HAART 1996-2004			Expanded Testing and Prevention 2005-2013			Modern Era: *Ending the HIV Epidemic* 2014-2024		
	Persons Tested (n)	HIV+ Cases (n)	Rate ^a^	Persons Tested (n)	HIV+ Cases (n)	Rate ^a^	Persons Tested (n)	HIV+ Cases (n)	Rate ^a^	Persons Tested (n)	HIV+ Cases (n)	Rate ^a^	Persons Tested (n)	HIV+ Cases (n)	Rate ^a^
Total	46,409,929	11,280	24.3	8,474,969	2,665	31.4	10,085,560	2,242	22.2	13,300,592	3,072	23.1	14,548,808	3,301	22.7
Sex															
Male	39,336,799	10,865	27.6	7,499,865	2,541	33.9	8,662,379	2,118	24.5	11,265,323	2,985	26.5	11,909,232	3,221	27.0
Female	7,073,130	415	5.9	975,104	124	12.7	1,423,181	124	8.7	2,035,269	87	4.3	2,639,576	80	3.0
Age group, y															
<20	5,465,222	548	10.0	1,051,461	95	9.0	1,405,051	95	6.8	1,391,009	178	12.8	1,617,701	180	11.1
20–24	14,368,318	3,787	26.4	2,769,793	810	29.2	3,184,365	612	19.2	4,142,734	1,080	26.1	4,271,426	1,285	30.1
25–29	9,277,994	2,944	31.7	1,625,310	777	47.8	1,813,500	477	26.3	2,778,136	751	27.0	3,061,048	939	30.7
30–34	6,415,196	1,789	27.9	1,158,514	487	42.0	1,354,238	456	33.7	1,735,649	367	21.1	2,166,795	479	22.1
35–39	5,041,910	1,188	23.6	851,094	276	32.4	1,156,359	352	30.4	1,429,227	322	22.5	1,605,230	238	14.8
> 40	5,841,289	1,024	17.5	1,018,797	220	21.6	1,172,047	250	21.3	1,823,837	374	20.5	1,826,608	180	9.9
Race and ethnicity															
White, non-Hispanic	28,777,369	3,419	11.9	5,752,788	1,059	18.4	6,240,806	667	10.7	8,466,968	903	10.7	8,316,807	790	9.5
Black, non-Hispanic	7,883,708	5,822	73.8	1,638,605	1,385	84.5	1,867,116	1,185	63.5	2,056,084	1,574	76.6	2,321,903	1,678	72.3
Hispanic	5,270,878	1,172	22.2	438,325	111	25.3	964,904	176	18.2	1,515,221	354	23.4	2,352,428	531	22.6
Other / unknown	4,477,974	867	19.4	645,251	110	17.0	1,012,734	214	21.1	1,262,319	241	19.1	1,557,670	302	19.4
Education level															
High school or less	32,362,591	8,883	27.4	6,579,109	2,279	34.6	7,685,461	1,841	24.0	9,149,428	2,349	25.7	8,948,593	2,414	27.0
Some college	4,455,128	920	20.7	365,225	87	23.8	558,327	114	20.4	1,534,288	299	19.5	1,997,288	420	21.0
Bachelor's or advanced degree	7,936,394	1,141	14.4	1,140,036	185	16.2	1,443,666	213	14.8	2,253,703	370	16.4	3,098,989	373	12.0
Other / unknown	1,655,816	336	20.3	390,599	114	29.2	398,106	74	18.6	363,173	54	14.9	503,938	94	18.7
Marital status															
Single, never married	22,508,360	7,554	33.6	4,071,124	1,717	42.2	5,061,705	1,461	28.9	6,101,069	2,115	34.7	7,274,462	2,261	31.1
Married	21,853,133	3,165	14.5	4,099,908	834	20.3	4,677,935	666	14.2	6,546,514	786	12.0	6,528,776	879	13.5
Other / unknown	2,048,436	561	27.4	303,937	114	37.5	345,920	115	33.2	653,009	171	26.2	745,570	161	21.6
Rank, grade															
Junior enlisted (E1–E4)	22,993,670	6,066	26.4	4,363,368	1,391	31.9	5,307,776	1,141	21.5	6,415,240	1,599	24.9	6,907,286	1,935	28.0
Senior enlisted (E5–E9)	16,691,404	4,432	26.6	3,010,732	1,135	37.7	3,436,665	939	27.3	4,927,008	1,215	24.7	5,316,999	1,143	21.5
Junior officer (O1–O3)	3,549,499	459	12.9	606,147	77	12.7	680,264	94	13.8	1,013,281	131	12.9	1,249,807	157	12.6
Senior officer (O4–O10)	2,539,134	273	10.8	379,670	52	13.7	517,698	59	11.4	774,314	108	13.9	867,452	54	6.2
Warrant officer (W01–W05)	636,222	50	7.9	115,052	10	8.7	143,157	9	6.3	170,749	19	11.1	207,264	12	5.8
Military occupation															
Combat-specific (Infantry / artillery / combat engineering / armor)	5,940,291	972	16.4	903,850	200	22.1	1,333,144	181	13.6	1,908,942	256	13.4	1,794,355	335	18.7
Motor transport	1,736,637	504	29.0	234,803	103	43.9	464,875	79	17.0	504,772	114	22.6	532,187	208	39.1
Pilot / air crew	1,505,453	112	7.4	199,079	16	8.0	318,645	28	8.8	493,510	36	7.3	494,219	32	6.5
Repair / engineering	10,821,213	2,166	20.0	1,575,310	362	23.0	2,295,425	397	17.3	3,318,343	622	18.7	3,632,135	785	21.6
Communications / intelligence	8,457,040	3,019	35.7	1,110,753	597	53.7	1,676,142	580	34.6	2,728,240	955	35.0	2,941,905	887	30.2
Health care	3,183,230	1,061	33.3	409,468	180	44.0	638,918	199	31.1	999,519	380	38.0	1,135,325	302	26.6
Other	14,766,065	3,446	23.3	4,041,706	1,207	29.9	3,358,411	778	23.2	3,347,266	709	21.2	4,018,682	752	18.7
Service branch															
Army	22,558,658	5,924	26.3	4,487,647	1,426	31.8	4,937,643	1,096	22.2	6,264,925	1,558	24.9	6,868,443	1,844	26.8
Marine Corps	6,087,759	879	14.4	1,143,998	174	15.2	1,662,547	201	12.1	1,641,934	238	14.5	1,639,280	266	16.2
Navy	11,472,225	3,479	30.3	2,839,304	1,065	37.5	3,393,677	916	27.0	2,614,522	797	30.5	2,624,722	701	26.7
Air Force ^b^	5,743,352	917	16.0	—	—	—	—	—	—	2,572,276	447	17.4	3,171,076	470	14.8
Coast Guard ^b^	547,935	81	14.8	4,020	—	—	91,693	29	31.6	206,935	32	15.5	245,287	20	8.2
Component															
Active	31,922,686	7,183	22.5	5,582,275	1,764	31.6	7,356,912	1,477	20.1	9,322,536	1,984	21.3	9,660,963	1,958	20.3
Guard	7,316,696	1,889	25.8	1,253,170	372	29.7	1,140,838	308	27.0	2,139,806	478	22.3	2,782,882	731	26.3
Reserve	7,170,547	2,208	30.8	1,639,524	529	32.3	1,587,810	457	28.8	1,838,250	610	33.2	2,104,963	612	29.1

Abbreviations: HIV, human immunodeficiency virus; HAART, highly active antiretroviral therapy; n, number; HIV+, HIV-positive; y, years; E, enlisted; O, officer.

a Rate per 100,000 persons tested.

b Due to incomplete or unavailable specimen and HIV testing results, data for Coast Guard are not available prior to 1996, nor for Air Force prior to 2006.

### Sex-stratified seropositivity rates

**FIGURE 2.**
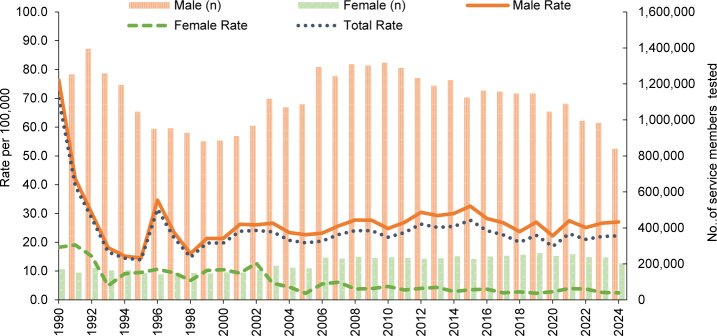
Total and Sex-Stratified HIV Antibody Seropositivity Rates, U.S. Armed Forces, 1990–2024

Male service members constituted an overwhelming majority, 96.3% (n=10,865), of all cases of HIV-antibody seropositivity documented during the surveillance period, with the total trend for the U.S. Armed Forces closely paralleling that observed for male service members **(Figure 2)** . The incidence rate among service men was highest in 1990, at 76.4 per 100,000 persons tested, before dropping to 14.5 (around 81% decline) in 1995, the lowest recorded in any year during the 35-year observation period. With the exception of a small spike in 1996, rates subsequently stabilized, averaging 25.7 new infections per year per 100,000 persons tested from 1997 through 2024. This average rate is much lower than the peak, but still higher than the recorded minimum.

**FIGURE 3.**
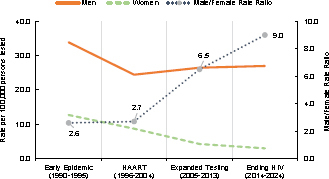
HIV Antibody Seropositivity Rates by Sex and Epidemic Phase

Seropositivity rates among female service members were much lower. The peak rate among women was observed in 1991, at 19.1 new infections per 100,000 tested persons, which declined sharply to 4.9 (74.4% decline) in 1993. Rates were relatively stable in subsequent years, fluctuating within a range of 6.7 to 12.7 per 100,000 tested persons until 2002, averaging 11.2 new cases per year. Starting in 2003, rates for women slowly but progressively declined, ultimately reaching one of their lowest levels, 2.5 cases per 100,000 persons tested (an approximately 87% decline), in 2024. The growing divergence between the sexes is further illustrated by the male-to-female rate ratios, which rose from a 3-fold difference during the earlier 2 phases to a 9-fold difference in the current phase **(Figure 3)** .

Aggregated rates of HIV seropositivity for the differentiated phases of the epidemic broadly reflect the annual rates. The highest seropositivity rate, 31.4 per 100,000 persons tested (n=2,665), was observed during the earliest phase of the HIV epidemic, which was followed by a 29.3% decline to 22.2 per 100,000 persons tested (n=2,242) in the second phase, defined by the introduction of HAART. The rate of decline slowed in subsequent phases, even increasing to 23.1 per 100,000 persons tested (n=3,072, 3.9% increase) in the third phase, which was characterized by expanded testing and prevention efforts, before decreasing slightly to 22.7 per 100,000 persons tested in the current phase (n=3,301), marked by the launch of Ending the HIV Epidemic initiative **(Table 2)** .

### Age and race-stratified seropositivity rates

Over 90% of HIV infections occurred among individuals under age 40 years **(Table 2)** . The lowest rate (10.0 per 100,000 persons tested) was observed in the youngest age group, under age 20 years, while the highest (31.7 per 100,000 persons tested) was among those in the 25-29-year age group. The trajectory of HIV seropositivity rates by age group also revealed diverging trends. Among individuals over age 30 years, rates steadily declined, ultimately decreasing by half during the past decade **(Table 2)** . While incidence rates among service members under age 30 years initially declined along with other age groups, the trend was not sustained, and in the current phase, aggregated rates for those under age 30 years either mimicked or exceeded those observed in the earliest phase.

**FIGURES 4a–4c.**
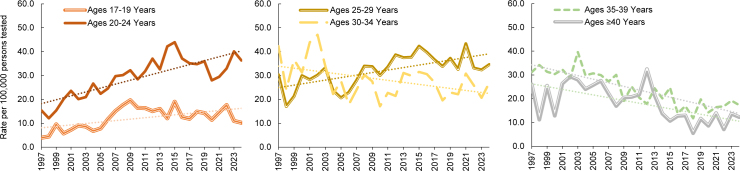
HIV Antibody Seropositivity Rates Among Male U.S. Service Members by Age Group and Year, 1997–2024

Age-stratified, annual trends for men show the lowest rates among the youngest age groups, with 4.0 cases per 100,000 persons tested in 1997 for those under age 20 years and 15.4 cases per 100,000 persons among those aged 20-24 years; by 2024, the rates for those age groups more than doubled, reaching 10.2 and 36.3 cases per 100,000 persons tested, respectively **(Figures 4a–4c)** . The older age groups (35-39, 40+) of male service members started at much higher levels, at rates of 31.2 and 26.1 per 100,000 persons tested, respectively, and their rates steadily declined over time, nearly halving to 17.3 and 12.2 per 100,000 persons tested, respectively, by 2024. The trends for the 2 intermediate age groups (25-29, 30-34) were less pronounced and overlapped, with consistently high rates for both groups throughout the period. The male 25-29-year age group evinced a weak upward trend, while the male 30-34-year age group started at the highest observed rate, 42.3 cases per 100,000 persons tested, in 1997 and decreased to 27.0 cases per 100,000 persons tested by 2024.

Despite representing 17.0% of those tested, non-Hispanic Black individuals accounted for more than half of all positive cases, with an overall rate of 73.8 per 100,000 persons tested **(Table 2)** . Non-Hispanic White individuals comprised 62.0% of all persons tested but had the lowest rate, 11.9 per 100,000 persons tested.

New diagnoses among non-Hispanic White service members steadily declined, from 18.4 in the earliest phase to 10.7 per 100,000 persons tested during the second and third phases, reaching a low of 9.5 per 100,000 persons tested in the last decade, a nearly 50% reduction **(Table 2)** . In contrast, the decline among non-Hispanic Black service members was less pronounced and consistent. Rates among Hispanic and Other or Unknown race and ethnicity categories remained largely unchanged.

**FIGURES 5a–5c.**
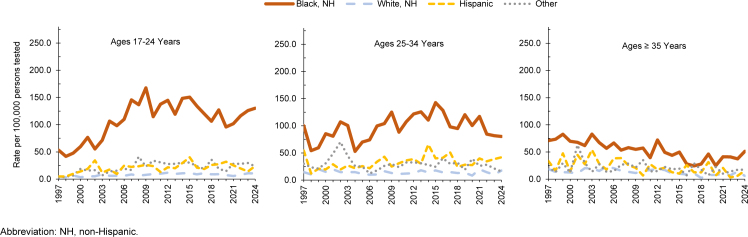
HIV Antibody Seropositivity Rates Among Male U.S. Service Members by Age Group, Race and Ethnicity, and Year, 1997–2024

HIV infection rates among male service members under age 25 years have risen among all ethnic and racial groups since 1997. **Figures 5a–5c** present annual HIV trends for male service members stratified by age as well as ethnic and racial group. The sharpest rise was observed among Hispanic men, with rates increasing from 2.4 in 1997 to 23.5 per 100,000 persons tested in 2024, a nearly 10-fold increase. Rates among non-Hispanic Black and White male service members under age 25 years more than doubled. Among non-Hispanic Black male service members, rates rose from 53.2 persons tested in 1997 to 130.3 in 2024, with a peak of 167.6 per 100,000 persons tested in 2009. Rates for non-Hispanic White male service members rose from 4.7 per 100,000 persons tested in 1997 to 10.4 in 2024, with a peak rate of 11.8 in 2012. Among those in the Other or Unknown race category, rates increased nearly 5-fold.

**FIGURES 6a–6c.**
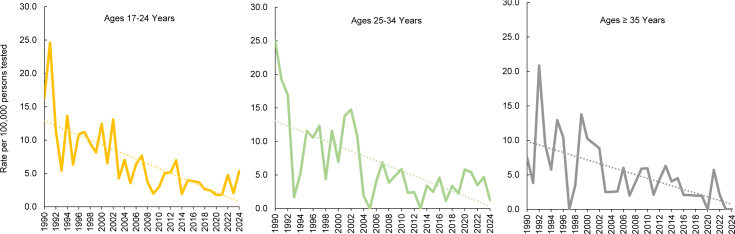
HIV Antibody Seropositivity Rates Among Female U.S. Service Members by Age Group and Year, 1990–2024

Due to low case numbers, annual female rate variability was high during the entire surveillance period, but there was an overall trend of consistent decline among all age groups. **Figures 6a–6c** present age-stratified, annual trends for female service members. In the earliest years of the surveillance period, female rates reached as high as 25 cases per 100,000 persons tested but gradually declined to about 5 cases or less per 100,000 persons tested among those under age 35 years. In recent years, no cases have been reported among service women over age 35 years. Similar to trends observed among their male counterparts, non-Hispanic Black female service members, overall, had the highest HV rates throughout the surveillance period and among all age groups (data not shown).

### Socio-economic and military characteristics

Service members with high school education or less comprised nearly 80% of HIV-positive cases. Although married individuals represented roughly half (47.1%) of all tested persons, they accounted for only 28.1% of positive cases **(Table 2)** . Enlisted service members had approximately twice the positivity rate of officers. Case distribution among occupational categories reflected, in general, that of the overall population, but personnel in communications / intelligence, health care, and motor transport exhibited higher rates of HIV seropositivity during all epidemic phases, at approximately 30 cases per 100,000 tested persons.

## Discussion

This report presents the results of HIV screening programs in the U.S. military from 1990 to 2024 within the broader context of the evolving HIV epidemic. Given the uniformity of care standards, robust screening protocols, and medical fitness requirements, this analysis of a 35-year surveillance period offers new insights into the current trajectory of HIV incidence among U.S. military personnel. ^1 , 9^ Recent policy changes, including the 2022 DOD policy affirming medically fit HIV-positive individuals' right to serve ^10^ and the 2024 court ruling ^11^ allowing accession by HIV-positive applicants, necessitate provision of the most up-to-date evidence to address the implications of these policies for HIV transmission and ensure HIV care and treatment programs are well adapted to support service members living with HIV.

Stratified analysis revealed significant differences in HIV-antibody seropositivity, by age, sex, and race, within the U.S. Armed Forces. New HIV diagnoses among male service members under age 30 years have steadily increased, with the greatest burden among non-Hispanic Black men and highest rise among Hispanic men. Although non-Hispanic White service members have the lowest recorded rates of new HIV diagnoses, rates for both non-Hispanic White and Black male service members under age 25 years more than doubled in 2024 compared to 1997.

Among Hispanic male service members men under age 25 years, the increase in HIV diagnoses was nearly 10-fold. This increase among Hispanic service members generally corresponds with national 2010-2022 data that show a 24% increase in HIV among the Hispanic population, during a period when the national HIV rate decreased by 12% overall. ^12 - 14^ The sharp rise in the number of HIV cases among Hispanic service members reflects both a growing share of Hispanics within the U.S. military and actual increases in infection rates that could be driven by behavioral and structural factors.

A recent study by Goodreau et al. that analyzed data from the American Men's Internet Survey found declining condom use and rise in condom-less sex among HIV-negative MSM not using PrEP, with the most substantial increase noted among Hispanic men aged 15-24 years. ^15^ This finding suggests that high-risk behavioral factors may be playing role in rising infection rates in Hispanic service members, as well as those of other racial and ethnic groups. Although DMSS does not explicitly collect data on same-sex behavior, studies assessing sexual risk behaviors among service members have shown that MSM represent a significant proportion of the population at high risk for HIV infection within the armed forces. ^16 - 18^

Geographic disparities appear to further influence HIV risk among men of color. The CDC reports that Hispanic or Latino individuals accounted for up 42% of new infections in the southern U.S., a region with historically higher HIV burden. ^14 , 19^ Similar trends were found in urban centers, with a multi-city study reporting increased HIV prevalence among MSM ages 23-29 years, from 10.2% in 1994-1999 to 16.7% in 2005-2011, with prevalence among MSM ages 18-22 years in Baltimore city nearly doubling, from 4.8% to 9.3%, during the same periods. ^20^ These findings emphasize the dynamic nature of HIV among young men in specific geographic areas and suggest that military HIV prevention programs should consider regional variations when designing prevention strategies.

The results of this study indicate increasing vulnerability of young male U.S. service members to HIV and suggest need for intensive and improved prevention strategies for this specific demographic, including condom use and healthy sexual behavior promotion. Shifting perceptions of HIV risk, including fading fear of HIV, reduced condom use, greater reliance on biomedical prevention such as PrEP, and “Undetectable=Untransmittable” messaging have been reported as factors potentially driving a rise in HIV among young adults in the general population. ^12 , 13 , 19^ These factors are likely contributing to rising infection rates among young male service members as well.

HIV PrEP has become a critical tool in HIV prevention, particularly among young men in the U.S. Studies show increased PrEP use among young men both nationally and within the military. ^21 , 22^ Reported PrEP usage disparities persist, however, with lower use among non-Hispanic Black and Hispanic men. ^23^ A 2023 National HIV Behavioral Surveillance (NHBS) report found that less than half of non-Hispanic Black and Hispanic MSM reported current PrEP use, which has been attributed to systemic and structural inequities, including barriers to health care access and cultural constraints. ^12 , 13 , 24^

The evolving dynamics of HIV antibody positivity rates in the U.S. military emphasize the need for continuous adaptation of prevention and screening strategies. While the overall trend shows a decline in HIV incidence, with the steepest drop occurring in the first decade of the screening program, total rates have plateaued since 1997. Increasing rates of HIV-antibody seropositivity among young, particularly under 25 years of age, male service members evidence a critical gap in HIV prevention efforts. By addressing behavioral shifts, improving PrEP accessibility, and incorporating demographic and regional risk factors into the design of intervention strategies, the U.S. military can strengthen its HIV prevention strategy and program, and safeguard the health and readiness of the force.
